# Self-reported vomiting during pregnancy in North-east Nigeria: perceptions, prevalence, severity and impacts

**DOI:** 10.1186/s12884-022-04916-4

**Published:** 2022-08-04

**Authors:** Judith Yargawa, Zelee Hill, Edward Fottrell

**Affiliations:** Institute for Global Health, University College London, London, UK

**Keywords:** Vomiting during pregnancy, Perceptions, Prevalence, Severity, Impacts

## Abstract

**Background:**

Vomiting is a common ailment during pregnancy, often linked to negative impacts on women’s quality of life. Very little is known about the issue in low income settings, particularly Sub-Saharan Africa, with studies from high income settings predominantly informing the evidence base. This study aimed to explore how women perceive vomiting during pregnancy and to measure its prevalence, severity and impacts in North-east Nigeria.

**Methods:**

Qualitative in-depth interviews, focus group discussions, family interviews and a cross-sectional household survey were carried out between December 2015 and November 2016 with women who had given birth within the past two years. Purposive sampling and thematic analysis were used in the qualitative studies. A three-staged cluster sampling with 640 women and descriptive analyses were used in the survey.

**Results:**

Women in the qualitative studies reported that vomiting was a normal part of pregnancy, unless a woman vomits after eating, has poor appetite, is not well-nourished, cannot perform chores, is overwhelmed by it or has to go to hospital. In the survey, 35.4% (95% CI 26.5–45.5) of women reported any vomiting during their last pregnancies and of these only 21.1% said it had stopped entirely within the first trimester. Over half of women who reported vomiting did so at least three times per day most days and 34.7% were vomiting five or more times per day during the most severe period. Care-seeking was reported by 61.5%. Both the qualitative and quantitative data found that vomiting impacted women in multiple ways including nutritionally, physiologically, mentally, financially and martially; 50.8% of women with any vomiting in the survey perceived the overall severity of the condition negatively.

**Conclusion:**

Vomiting during pregnancy is dually seen as normal and problematic depending on its characteristics and impacts. The burden appears to be high with many women seeking care for the condition.

**Supplementary Information:**

The online version contains supplementary material available at 10.1186/s12884-022-04916-4.

## Background

Nausea and vomiting of pregnancy (NVP) is one of the most commonly experienced morbidities during pregnancy with prevalence estimates ranging from 35 to 91% [1]. It has been linked to adverse effects on quality of life, mental health, ability to carry out day-to-day tasks, economic productivity and women’s willingness to become pregnant again [2–10]. It is a condition of public health importance given its connection to nutrition during pregnancy, impacts on women’s lives and resource implications on health services [2–7, 11, 12]. Hyperemesis gravidarum, the most severe form of vomiting, affects about 0.3- 3.6% of pregnancies [1] and can be life-threatening without medical intervention. Hyperemesis gravidarum is the most common reason for hospitalisation in the first half of pregnancy in some settings [13] and incurs significant financial costs for families and the health service [14]. It has also been linked to mental health conditions [15, 16].

As maternal mortality declines, the global agenda on maternal health has gradually shifted to the burden and impacts of maternal morbidity, including less severe or non-life threatening ones. NVP is included in the Maternal Morbidity Matrix [17], arguably the most comprehensive tool for measuring maternal morbidity, which was developed by a World Health Organisation technical working group of experts from low, middle and high income countries [18]. Very little, however, is known about vomiting in low income settings as studies from high income settings overwhelmingly inform the evidence base. Of 59 studies included in a systematic review and meta-analysis on global NVP rates, only two studies were from Sub-Saharan Africa and these were published approximately three decades ago [1].

In addition to sparse quantitative data, our initial scoping review found no studies on women’s perceptions of vomiting during pregnancy nor their lived experiences in Sub-Saharan Africa, whereas such reports are well-documented in high income settings [2–7]. It is thus important to obtain data from this underrepresented part of the globe to improve the evidence base. This mixed-methods study aimed to explore and measure vomiting during pregnancy within communities in Yola, North-east Nigeria. Its specific objectives included: to find out women’s perceptions of vomiting during pregnancy; to estimate the prevalence of self-reported vomiting; to measure the severity of the vomiting; and to investigate the impacts of vomiting during pregnancy. The paper focuses on vomiting as opposed to nausea and vomiting. While separating these two conditions may appear arbitrary, we were mainly interested in researching about vomiting as it emerged as particularly important to women in a pre-pilot study that we conducted and it is also rarely studied in low income settings.

## Methods

The methods for this study have been described in detail elsewhere [19]. In brief, the study was part of a two-phased research project on maternal morbidity within communities in Yola, North-east Nigeria. Yola is the capital of Adamawa State with a population of 823,220 [20]. There are two Local Government Areas in Yola: Yola North, which is urban; and Yola South, which has both urban and rural areas. In the 2018 Nigeria Demographic and Health Survey, only 20.7% of women aged 15–49 years in Adamawa state had completed secondary school, with about half (47.0%) having no education [21]. While the proportion of women who received skilled antenatal care is high at 82.1%, only 40.5% of women gave birth with a skilled attendant [21]. Qualitative studies- focus group discussions (FGDs), in-depth interviews (IDIs) and family interviews- were carried out to explore women’s perceptions of vomiting during pregnancy as well as its impact on their day-to-day lives. These were followed by a cross-sectional household survey to measure the prevalence, severity and consequences of the condition, all conducted in 2015–2016. Eligible women were married residents of Yola aged 15–49 years who had given birth within two years preceding the study. Respondents aged 15–17 years old were included as it is not uncommon for women in that age bracket to be married and have children in some communities in the study area. Three different sets of law (civil, Islamic and customary) run concurrently in the country, hence some states with Islamic and customary laws allow girls below age 18 to marry [22–24].

In the qualitative studies, respondents were recruited through community liaisons, a women’s empowerment community centre, and snowball sampling. Respondents were sampled purposively to give a range of ages, educational levels, rural/urban residences and self-reported morbidity experiences. For each sub-group, estimated sample sizes were developed in sampling grids but data collection continued until saturation. Semi-structured topic guides were used and data were collected in Hausa or English based on the respondent’s fluency. In the IDIs, the woman was asked whether she vomited at any point during her pregnancy, and if so, the frequency, whether the vomiting was such that almost everything that goes into her mouth comes out, whether or not she thought this was normal or an illness and how the experience impacted her (Additional file 1). In the FGDs, we did not explore individual cases of vomiting but rather asked about general perceptions and beliefs, for example, women were asked whether they thought vomiting was an illness or a normal part of pregnancy (Additional file 1). The family interviews were conducted with husbands, co-wives or other females in the women’s social circles who played substantial roles in their maternal health phase; these were mainly conducted to explore care-seeking for health problems and were included in this study where the health problem related to vomiting. All sessions were audio-recorded and transcribed, except the family interviews which were analysed directly from the audio-recordings as they contained few relevant data. Using thematic analysis [25], data were analysed both inductively and deductively and managed using NVivo 10. Direct quotes have been reported using pseudonyms.

In the household survey, a three-stage cluster sampling at the ward, settlement and respondent levels was carried out using probability proportional to size; the sampling frame with population sizes was obtained from local authorities. A sample size of 660 was obtained from calculations based on parameters, including a 1.5 design effect and taking into account a 10% non-response rate. Using the Expanded Programme on Immunisation method [26, 27], 11 respondents were selected from each of 60 clusters (settlements) in total. A paper-based questionnaire was administered face-to-face in English of Hausa by female data collectors. Women were asked whether they vomited more than two times per day at any point during their pregnancy even if this did not continue throughout the pregnancy. Respondents were asked about duration of the vomiting and how many times they vomited per day most of the time during this period. They were also asked about the number of vomiting episodes per day at the most severe period of the vomiting (Additional file 2). We collected data on severity relating to the respondent’s reports of their ability to retain food, weight loss, whether the vomiting made them afraid, whether they thought they were going to die and care-seeking. To measure the impacts of vomiting on different aspects of respondents’ lives (physical, restrictions at home imposed, marital, social), a number of statements were read to them with likert responses: ‘strongly agree’, ‘mildly agree’, ‘mildly disagree’, ‘strongly disagree’ (Additional file 2); a facial expression card was used to facilitate comprehension [28]. An additional question asked respondents to indicate the overall severity of the pain/discomfort/distress of the vomiting and the Facial Affective Scale (FAS), a tool originally designed to measure pain in children [29] was used as an enablement tool. EpiData 3.1 was used for data entry and the data were analysed descriptively using Stata 14, with weights and adjustments applied accordingly.

## Results

### Characteristics of respondents

In the qualitative studies, seven FGDs (with five to eight women per FGD, 44 women in total), 21 IDIs and 10 family interviews were carried out. The FGD and IDI respondents’ ages ranged from 15- 48 years. Thirty-four out of 44 FGD respondents and 14 out of 21 IDI respondents had no/primary education. Place of delivery was varied: 13 women in the IDIs and most women in the urban FGDs had health facility deliveries, while the rural FGDs were nearly split evenly between health facility and home births. Regarding residence, 11 and 20 women in the IDIs and FGDs respectively lived in rural areas. In the family interviews, four houses were located in urban areas and six households were in rural areas.

We identified five cases that we classified as moderate or severe vomiting in the IDIs based on respondents’ descriptions of its impact on their lives and biomedical symptoms consistent with the health problem [30]; all other IDI respondents reported mild or no vomiting. Although there were only five cases of moderate or severe vomiting, these offer important insights into what it is like to experience this condition in a low income setting.

In the cross-sectional study, 640 women participated (97% response rate). Of these, 222 women or 35.4% (95% CI 26.5–45.5) reported that they vomited at least twice a day at some points during their last pregnancies; their socio-demographic characteristics have been summarised in Table 1 (see Additional file 3 for data on the entire participant population). Over 70% of the respondents who vomited lived in urban areas, were aged 20–34 years and were Muslim, with the general participant population showing a similar distribution (Additional file 3). A little over half of the women were unemployed/house-wives (57.7%) and could not read in any language (55.7%). Only 8.3% of the respondents had post-secondary education.

**Table 1:** Characteristics of survey respondents who reported vomiting during pregnancy

Characteristic	Frequency	Weighted Proportion % (95% CI)
**Residence**		
Rural	39	26.0 (8.4- 57.6)
Urban	183	74.0 (42.5- 91.6)
**Age (years)**		
15–19	18	8.1 (5.1- 12.6)
20–34	168	76.9 (74.1- 79.5)
35–49	33	15.0 (10.8- 20.4)
**Type of marital union**		
Monogamous	176	75.8 (68.4- 81.9)
Polygamous	43	24.2 (18.1- 31.6)
**Religion**		
Islam	179	74.2 (58.2- 85.5)
Christianity	43	25.9 (14.5- 41.8)
**Literacy**		
Can read in any language	96	44.3 (35.1- 54.1)
Cannot read in any language	114	55.7 (46.0- 65.0)
**Main occupation**		
Unemployed/house-wife	130	57.7 (53.6- 61.7)
Unskilled	63	31.9 (24.9- 39.9)
Skilled	27	10.4 (6.4- 16.3)
**Highest educational level completed/currently attending**		
Never attended school/ non-western education	60	31.7 (22.6- 42.4)
Primary	50	19.7 (15.5- 24.7)
Secondary	91	40.3 (30.9- 50.6)
Post-secondary	21	8.3 (4.7- 14.3)
**Husband’s main occupation**		
Unemployed	3	1.8 (1.1- 3.1)
Unskilled	125	59.2 (49.0- 68.7)
Skilled	94	39.0 (29.6- 49.2)
**Husband’s highest edu. level completed/currently attending**		
Never attended school/ non-western education	39	23.9 (17.2- 32.0)
Primary	14	7.6 (5.2- 11.0)
Secondary	94	38.9 (34.1- 44.1)
Post-secondary	70	29.6 (21.6- 39.2)

* Numbers may not add up due to missing data

### How vomiting during pregnancy is perceived

Most respondents in the qualitative studies generally perceived vomiting as a normal part of pregnancy, unless a woman vomits after eating, has poor appetite or is not well-nourished as a result of the vomiting. They provided diverse descriptions of vomiting during pregnancy, which we have classified as ‘normal’ and ‘abnormal’ vomiting. ‘Normal’ vomiting is short, that is one feels uncomfortable for few hours a day, or it only occurs ‘*once in a while.’* It does not prevent one from performing chores, or induce the need to lie down, and has triggers that can be controlled such as avoiding food odours. In contrast, ‘abnormal’ vomiting is ‘*overwhelming,’* causes one to vomit everything she eats/drinks, bad enough to go to hospital and is prolonged- defined as vomiting from ‘*the moment pregnancy sets in…until you give birth’*, vomiting beyond the first trimester, or vomiting for a significant duration of the pregnancy. A few women mentioned that vomiting “*varies from pregnancy to pregnancy,”* or “*depends on the individual; someone will experience it, another person will not*.” The women who experienced moderate or severe vomiting tended to perceive it as ‘abnormal’ or an illness, as seen below:

*Respondent: Yeah some women think that all those nausea, vomiting is normal but to me it’s not normal because I have seen so many pregnant women that from Day 1 they are eating like pig until they deliver… To me it’s not normal when it [vomiting] can deprive you of eating what your body needs you know, or maybe eating what you are supposed to eat, to me it’s not normal. But some people think that all those nausea, vomiting is normal…But I don’t think I would call it normal even what they call normal symptom…I wouldn’t call it normal because I [am]supposed to take those things I need them and I can’t because once I take them I vomit so…* (IDI 13, urban, post-secondary education, parity 3, moderate/severe vomiting group).

### Severity of the vomiting cases

In the IDIs, the women who had experienced moderate or severe vomiting reported being unable to keep food and even water down. They reported vomiting ‘several times’ up to five times in a day. Two women had severe cases and mentioned that they were vomiting from the first trimester until birth; they received drips and one was hospitalised. The moderate cases had shorter duration. When one of the IDI respondents was asked how she survived the long period of being unable to retain food and water for months, she mentioned that “*it was God who sustained me”* and also assumed that “*no matter how difficult it was, there will still be some [food] that will hang in there from the one I ate and then vomited*” (IDI 5).

The survey showed similar accounts of severity (Table 2). Amongst all women who vomited more than two times per day even if this did not continue to the end of the pregnancy, over half of the women vomited at least three times per day most days when they were vomiting, and 16.1% vomited five or more times per day. During the most severe periods, 76.7% of women vomited at least three times a day and 34.7% five or more times a day. For this latter group of women, 75.3% reported that this severe period had lasted for three months or more.

**Table 2:** Severity of respondents’ vomiting experiences during pregnancy (n = 222)

Domain	Characteristic	Frequency	Weighted Proportion % (95% CI)
**Duration and episodes**	Duration		
	Started and ended in 1^st^ trimester	48	21.1 (13.5- 31.6)
	Started in 1^st^ trimester, ended in 2^nd^ trimester	88	39.4 (33.1- 46.2)
	Started in 1^st^ trimester, ended in 3^rd^ trimester	60	30.1 (19.5- 43.3)
	Other durations	20	9.3 (4.1–18.8)
	Vomiting episodes per day- most times		
	1–2 times	94	41.7 (30.3- 54.1)
	3–4 times	82	42.2 (31.1- 54.1)
	≥ 5 times	36	16.1 (11.5- 22.1)
	Vomiting episodes per day- most severe period		
	1–2 times	57	23.3 (13.5- 37.0)
	3–4 times	82	42.0 (29.0- 56.3)
	≥ 5 times	76	34.7 (23.3- 48.1)
	Duration of vomiting 3–4 times a day		
	Less than 1 week	2	3.9 (0.9- 15.0)
	1 week- 1 month	16	24.3 (14.4- 38.0)
	> 1 month but < 3 months	22	25.2 (15.1- 39.1)
	≥ 3 months	42	46.6 (30.1- 63.9)
	Duration of vomiting ≥ 5 times		
	Less than 1 week	1	1.2 (0.1- 9.8)
	1 week- 1 month	7	8.2 (3.7- 17.5)
	> 1 month but < 3 months	14	15.3 (8.2- 26.7)
	≥ 3 months	54	75.3 (60.8- 85.7)
**Symptoms and personal assessments**	Inability to retain food in stomach	156	73.4 (60.2- 83.5)
	Vomiting made her afraid	70	31.9 (21.2- 44.9)
	Thought she was going to die from the vomiting	53	23.0 (14.7- 34.2)
	Lost weight because of the vomiting	116	55.7 (39.1- 71.0)
**Care-seeking**	Care-seeking (women who sought care/treatment/remedy = 125 or 61.5%)		
	Home remedy/self- treatment	15	11.6 (5.4- 23.2)
	Consulted lay source	3	2.0 (0.4- 9.6)
	Consulted traditional source	1	0.7 (0.1- 5.9)
	Visited pharmacy	25	19.7 (11.0- 32.7)
	Summoned health worker home	6	6.1 (2.2- 16.1)
	Visited formal health facility	70	57.8 (48.2- 66.9)
	Treatment received		
	None	18	11.5 (6.3- 20.3)
	Conventional medicine/therapy	106	84.7 (73.8- 91.6)
	Traditional medicine/therapy	3	1.9 (0.4- 9.3)
	Other	8	5.1 (2.5- 10.3)
	Ever given drip for the vomiting (n = 43 or 24.4% of women vomiting)		
	1 drip	13	30.9 (18.6- 46.6)
	2–3 drips	14	30.9 (19.2- 45.8)
	≥ 4 drips	15	38.2 (22.0- 57.5)

* Numbers may not add up due to missing data. For “care-seeking options” and “care-seeking treatment received”, all options that applied were ticked and proportions were calculated per option

As shown in Table 2, women reported a variety of problems and fears related to their vomiting including an inability to retain food (73.4%), weight loss (55.7%), being afraid (31.9%) and fear of dying (23.0%). Care-seeking was reported by 61.5% of the respondents, with health facility and pharmacy visits dominating. Majority reported that they had received biomedical treatment but one in every 10 respondents (11.5%) reported not receiving any.

### Impacts of vomiting

The data on impacts of vomiting mainly came from the IDIs, primarily from the five women who had experienced moderate or severe vomiting; however, respondents from the FGDs, family interviews and the remaining IDIs also described potential consequences of more severe vomiting and the impact they had seen in others. The five women described how the vomiting impacted them nutritionally and physiologically. They could only keep certain foods down and went through months of their pregnancies restricted to specific foods and drinks such as tea, oranges, and *talge,* a pudding made with mainly maize or guinea-corn flour and water. Two of the women were totally unable to retain food or water at some points and had to be put on drips. All five women reported that they lost weight considerably: “*I was almost starving … I lost appetite and I was getting underweight you know, and the baby was just growing*” (IDI 13). Others reported physiological consequences including fainting and almost needing a blood transfusion during delivery as a result of poor nutrition.” One respondent felt that the vomiting was so severe she may die:

*Respondent: …Well, I said “This illness that has really disturbed me. If I will survive, let me survive; if it is for death, I have forgiven everyone and people should also forgive me” ...*

*(Respondent’s mother-in-law, who was nearby, interjects****)****: She thought she was going to die…That’s why she said everyone should forgive her… That’s how pregnancy is; it puts an individual into all sorts of things* (IDI 5, rural, no education, parity 4, moderate/severe vomiting group).

As well as nutritional and physiological consequences, severe or moderate vomiting had logistical implications for their families. As described above, abnormal vomiting was often defined by impact and women reported their families having to cook two separate meals because they could not tolerate the general meal; cooking in another house; and family members not wearing sprays or perfumes. The vomiting also inhibited the respondents from performing chores, making them fully dependent on family members. For the women who were given drips, their families had to pay out-of-pocket for their treatments. One respondent who reported that “*every time- almost all the time they were coming to add more water [for me at home] … I consumed many bags [drips]*” also provided the account below:

*Respondent: Sincerely we were spending money. Money, he [husband] was really spending a lot of money, honestly. Honestly, money was being spent… Some people even said that it was as if I was buying the children whenever I got pregnant... But I said, “No, it is not like that.” They said, “This kind of stress that you go through.” They said, “It is not everyone that can keep you with all this kind of dark suffering.” Did you see the way I used to change? It was likeeeeee a rag when you come and see me lying down. You might even say it is an ooooooold woman, I am just soooo folded, even to get up I can’t* (IDI 9, rural, some primary education, parity 3, moderate/severe vomiting group).

The vomiting also had an impact on family relationships with the husband of the respondent with the quote directly above suggesting family planning so that she could rest from the stressful vomiting experience. He also complained about having to “*scout around*” for someone to cook for him:

*Respondent: My husband, the situation even affected him. He even said that if there was a way to do it, that after this pregnancy when I give birth, he would prefer that I go to the hospital and get an injection so that I can take this break and rest. Because for him, kai this thing is really stressful. I said, “No. What God has given you would you tell him it’s not supposed to be so or how?” Then he said, “It is not like I am refusing it, it is the suffering that I want to prevent for you. When you get pregnant, to say that you cannot do anything, you’re just lying down and then I have to go and scout around for a woman to come and cook for me?”* (IDI 9, rural, some primary education, parity 3, moderate/severe vomiting group).

**Fig. 1:**
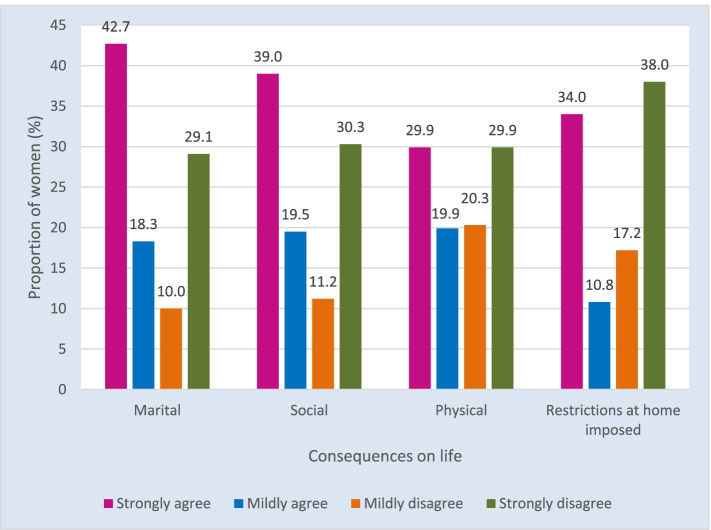
Proportion of women and their levels of agreement to statements on impacts of vomiting on different aspects of life (n = 222)

Figure 1 shows the proportion of women reporting impacts of vomiting on these aspects of their lives in the survey: 42.7% of women strongly agreed with the statements around marital consequences, 39.0% social, 34.0% restrictions at home imposed and 29.9% physical consequences. Including women who “mildly agreed” increased reporting of consequences to 61.0% (marital), 58.5% (social), 44.8% (restrictions at home imposed) and 49.8% (physical). Using the Facial Affective Scale [29], 50.8% of women perceived the overall severity of the vomiting negatively.

## Discussion

In the qualitative phase, vomiting was perceived as a normal part of pregnancy unless a woman is unable to retain anything ingested, vomits after eating, has poor appetite, isn’t well-nourished, has to go to the hospital, is overwhelmed by the vomiting, or experiences prolonged vomiting. Women also reported how the vomiting negatively impacted them and their families. The quantitative results showed that many women experienced vomiting during pregnancy (35.4%) and also vomited for prolonged periods. In addition, over half of these women vomited at least three times per day most times during the time in the pregnancy that they were vomiting and approximately three-quarters vomited at least three times per day at the most severe period (75.3% of those who had severe vomiting reported that this severe period had lasted for three months or more). High proportions reported negative consequences (Fig. 1) and half of the women (50.8%) perceived the overall severity of the vomiting negatively. These results highlight the prevalence and impacts of vomiting during pregnancy in this population.

The prevalence of vomiting shown in our study—35.4% (95% CI 26.5- 45.5) – is lower than estimates reported in other studies. In general medical literature, it is often reported that NVP affects around 70–80% of pregnant women [30]. Lakew et al. (2015) study’s in Ethiopia found that 47.1% of women had experienced nausea/vomiting [31] while Agampodi et al. (2013) found that 69.7% of the women they surveyed in a Sri Lankan district experienced NVP [11]. A meta-analysis found the global prevalence of NVP to be 69.4%, although most of the studies came from high income settings [1]. The estimate in our study may have been lower because we only focused on vomiting as opposed to nausea and vomiting. Separating these two conditions may appear arbitrary, but we were mainly interested in researching about vomiting because it emerged as particularly important to women in a pre-pilot study we undertook and it is also rarely studied in low income settings. In addition, the threshold that we imposed in defining vomiting (more than two times per day even if this did not continue to the end of the pregnancy) may have resulted in lower prevalence, as these other studies may have measured any occurrence of vomiting. We imposed the threshold to distinguish between occasional and consistent vomiting episodes.

The impacts of vomiting on women’s lives were prominent in our study, as seen in both the qualitative reports and survey. Vomiting seems to have a synergetic power to disrupt multiple aspects of women’s day-to-day lives. Physically, it prevented them from performing their activities/chores and increased the workload for their families. Nutritionally, it may have negatively impacted nourishment since 73.4% of the women who vomited reported being unable to retain food in the stomach. Logistically, it brought about structural changes for their families such as needing to cook in another house or cooking two separate meals. It also had physiological, marital, financial and mental health consequences (31.9% reported that the vomiting made them afraid and 23.0% said they vomited so much that they thought they would die). These negative impacts appeared to have involved the day-to-day lives of women and their families. Historically, non-severe conditions such as vomiting during pregnancy have received very little attention in low income settings due to more pressing maternal health issues in these regions. However, our findings show that these issues also require attention and it is encouraging to see the inclusion of vomiting in the Maternal Morbidity Matrix [17]. Key interventions and appropriate health messages should be provided during antenatal care in order to improve women’s quality of life and health during pregnancy. A surprise finding is the high proportion of women experiencing mental health consequences due to vomiting, an area deserving further research.

Given the importance of nutrition during pregnancy, it was striking that even in severe cases there was no evidence that these women replenished the food whenever they vomited. The women also reported needing to restrict their diets to control the vomiting, as studies in Ethiopia and the UK also found that women reduced their food intake as a coping mechanism against vomiting [32, 33]. It is worth mentioning that many women may have started pregnancies with a nutritional deficit as the staple food in Nigeria is mainly cereals [34]. Although vegetable-containing soups are sometimes eaten with these cereal-based food, consumption of fruits and raw vegetables are still quite low in Nigeria and are considered as ‘luxury goods’ in some quarters [35–37]. Undernutrition at a national level in women of reproductive age is 11% whereas overnutrition is 25% [38], with malnutrition inequalities higher among the least educated households, northern states and the Hausa ethnic group in northern Nigeria [21, 39]. Therefore, a pre-existing nutritional deficit coupled with the potential loss of nutrients from vomiting could worsen women’s nutritional status during pregnancy. In spite of its public health importance, the impact of vomiting on nutrition is still not clear in literature although Mohamadi et al. (2020) found an association between NVP and anaemia [40]. This is an area where more evidence is needed.

It is difficult to compare our findings on severity and impacts of vomiting to other Sub-Saharan African countries as we located no other studies from this setting. However, in Sri Lanka, Agampodi et al. (2013) asked women to report any illness episodes during pregnancy (with subsequent validation with medical records and diagnosis cards) and then they measured the effects of the morbidities on daily life using a visual analog scale [11]. They found the impact of NVP to be significant: it accounted for the highest proportion of hospitalisations (43.1%) amongst all morbidities reported in their study and also the highest level of total incapacitation and severe inhibition of every-day activities (32%) [11]. In as much as physical symptoms can highlight the debilitating impacts of vomiting, it is key to also consider non-physical impacts of vomiting. One study (although conducted in a high income setting) found that physical symptoms were weakly correlated with women’s self-assessment of the severity of their NVP, with the frequency of vomiting accounting for only 9% of the variability of their perceptions of severity (r^2^ = 0.09) [41]. They concluded that the severity that women feel cannot be described by the physical symptoms of the NVP alone, as the women considered their wellbeing overall and how other aspects of their lives were being affected. Our data support this finding and show the importance of considering impacts holistically beyond the frequency of the vomiting.

Our study had several strengths. It reports on a condition that is hardly researched in low income settings; therefore, contributing to filling important gaps in the evidence base on vomiting during pregnancy. It has also helped in directing some attention to the burden and impacts of vomiting so that care givers and other relevant groups can better serve women’s health needs. We used several methods to meet the study’s objectives, which allowed for triangulation especially relating to the severity and impacts of vomiting during pregnancy. However, we used non-validated self-reports of vomiting, a method with potential for recall and reporting bias especially given the relatively long recall period, although alternative methods to asking the women themselves are limited. Scores for measuring NVP exist [42, 43], however these may not have been valid for use throughout our setting due to the requirement to estimate number of hours of episodes per day, although this requires further exploration. We did not explore treatment regimens for vomiting during pregnancy in our study in-depth but this is one area that could provide additional insights. One systematic review found that a major reason for pregnant women’s use of medicinal plants in Africa was to relief NVP [44]. A Cochrane review also found a wide variety of interventions for NVP from ginger to lemon oil to acupuncture and antiemetic medications, with the authors suggesting the need for clear guidelines to health professionals and women on safe and effective interventions [45]. A U.S. study found increasing use of marijuana over time to relieve NVP [46]. It would be interesting to obtain perspectives on treatments for vomiting during pregnancy from low income settings, as these regimens may likely affect health. The impacts of vomiting on nutrition as well as its association with anaemia need further studies.

## Conclusions

We found a high prevalence of self-reported vomiting during pregnancy- one in every three women- and also found reports of negative impacts of the condition on women’s lives. Vomiting appears to be a significant issue in the setting, hence efforts should be geared towards improving the quality of life and health of women during pregnancy. More studies are needed on vomiting during pregnancy in low income settings since these appear to be largely non-existent, although the inclusion of vomiting in the recently developed Maternal Morbidity Matrix will hopefully raise its profile.

## Supplementary Information

**Additional file 1. **Questions asked on vomiting during pregnancy in in-depth interview and focus group discussion guides.

**Additional file 2. **Survey questionnaire.

**Additional file 3. **Entire participant population in survey (n=640).

## Data Availability

The datasets generated and/or analysed during the current study are not publicly available given the risks to anonymity and qualitative design but are available from the corresponding author on reasonable request.

## Abbreviations

CI: Confidence interval
FAS: Facial Affective Scale
FGD(s): Focus group discussion(s)
IDI(s): In-depth interview(s)
NVP: Nausea and vomiting of pregnancy
