# Supplementary material for: Self-reported vomiting during pregnancy in North-east Nigeria: perceptions, prevalence, severity and impacts
Source: BMC Pregnancy Childbirth. 2022 Aug 4;22:614. doi: 10.1186/s12884-022-04916-4 (PMC9351193; doi:10.1186/s12884-022-04916-4)
Supplement: Supplementary file 1 — Additional file 1. Questions asked on vomiting during pregnancy in in-depth interview and focus group discussion guides. [file 12884_2022_4916_MOESM1_ESM.docx]

## **Additional file 1: Questions asked on vomiting during pregnancy in in-depth interview and focus group discussion guides**

**In-depth interview questions**

Were you vomiting at any point during your last pregnancy? If yes: Tell me more it. Note:

- Its frequency
- Her weight at this period
- Was the vomiting such that almost everything that goes into her mouth comes out?

Ask these if you suspect HG (vomiting several times a day and weight loss may indicate HG)

- Did you think you were sick or you thought this was normal for a pregnant woman?
- What do you think may have caused this excessive vomiting?
- Did you consult any source about the excessive vomiting?
- How was this experience for you? What was it like to be vomiting this way? Please tell me how a typical day was for you during this time.

**Focus group discussion questions**

What do you think about vomiting during pregnancy? Do you think vomiting is an illness or ‘normal’ part of pregnancy? (Probe- context in which it is normal and when an illness).

Note: As this study was part of a bigger project on maternal morbidity within communities in Yola, North-east Nigeria, only questions relevant to this paper’s focus have been provided.
