# Supplementary material for: Self-reported vomiting during pregnancy in North-east Nigeria: perceptions, prevalence, severity and impacts
Source: BMC Pregnancy Childbirth. 2022 Aug 4;22:614. doi: 10.1186/s12884-022-04916-4 (PMC9351193; doi:10.1186/s12884-022-04916-4)
Supplement: Supplementary file 2 — Additional file 2. Survey questionnaire. [file 12884_2022_4916_MOESM2_ESM.docx]

## **Supplementary file 2: Survey questionnaire**

| VOMITING (VM) | | | | |
| --- | --- | --- | --- | --- |
| **S/N** | **Question** | **Response** | **Code** | **Go to** |
| VM1 | I would like to ask some specific questions about vomiting in your last pregnancy.  Were you vomiting frequently during your last pregnancy, that is, vomiting more than 2 times per day even if this did not continue to the end of the pregnancy? | Yes  No | 1  2 | VM2  PD1 |
| VM2 | When did the vomiting start? | First trimester  Second trimester  Third trimester  Don’t know | 1  2  3  99 |  |
| VM3 | When did the vomiting stop entirely? | First trimester  Second trimester  Third trimester  Don’t know | 1  2  3  99 |  |
| VM4 | Did you vomit so much that almost everything that goes into your mouth comes out? | Yes  No | 1  2 |  |
| VM5 | Did you vomit so much that you were afraid? | Yes  No | 1  2 |  |
| VM6 | Did you vomit so much that you thought you were going to die? | Yes  No | 1  2 |  |
| VM7 | Did you lose weight around this time that you were vomiting? | Yes  No  Don’t know | 1  2  99 | VM8 |
| VM8 | How did you know that you lost weight?  TICK ALL THAT APPLY | Clothes felt loose on body  Looked lean/ collar bones showed  Measured with tape- Dimensions narrower than before  Measured with scale- weighed less than before  Other | 1  2  3  4  5 |  |
| I am going to read some statements to you about your vomiting experience during your last pregnancy. Tell me whether you strongly agree, mildly agree, mildly disagree or strongly disagree with the statement. SHOW CARD. | | | | |
| VM9  VM10  VM11  VM12  VM13  VM14 | “The vomiting made me fully dependent on others to do my day-to-day activities like cooking, sweeping and going to the shop.”  “The vomiting was so serious that we restricted the usage of substances with distinct smell in my family, such as perfume and some cooking oil to avoid triggering the vomiting.”    WITH OCCUPATION ONLY: “The vomiting affected my occupation negatively such as making me to be absent from work, receiving reprimand(s) from my supervisor or missing opportunities to make money.”  STUDENTS ONLY: “The vomiting affected my studies negatively such as making me to be absent from class or missing tests/examinations.”  “The vomiting affected my relationship with my husband negatively such as making us quarrel, making us not to spend time together or making us not to be in good terms”  “The vomiting affected my social life negatively such as preventing me from visiting family and friends or making me to avoid gatherings.” | Strongly agree  Mildly agree  Mildly disagree  Strongly disagree  Strongly agree  Mildly agree  Mildly disagree  Strongly disagree  Strongly agree  Mildly agree  Mildly disagree  Strongly disagree  Strongly agree  Mildly agree  Mildly disagree  Strongly disagree  Strongly agree  Mildly agree  Mildly disagree  Strongly disagree  Strongly agree  Mildly agree  Mildly disagree  Strongly disagree | 1  2  3  4  1  2  3  4  1  2  3  4  1  2  3  4  1  2  3  4  1  2  3  4 |  |
| VM15 | Did you seek care/ treatment/ remedy for the vomiting, that is, any solution to the vomiting from anywhere or anyone? | Yes  No | 1  2 |  |
| VM16 | What did you do?  TICK ALL THAT APPLY | Home remedy/self-treatment  Consulted lay source (e.g. mum)  Consulted traditional source  Visited chemist  Summoned health worker home  Visited formal health facility | 1  2  3  4  5  6 |  |
| VM17 | What treatment did you receive?  TICK ALL THAT APPLY | None  Western medicine/ therapy  Traditional medicine/ therapy  Other alternatives | 1  2  3  4 |  |
| VM18 | Were you ever given a drip for the vomiting? | Yes  No  Don’t know | 1  2  99 | VM19  VM20 |
| VM19 | How many drips were you given throughout your pregnancy for the vomiting? | 1 drip  2-3 drips  4-5 drips  6 and above | 1  2  3  4 |  |
| VM20 | Did you pay for care (medical services) and/or treatment (medicines) for the vomiting? | Yes  No | 1  2 |  |
| VM21 | Did you or your family do any of the following to pay for care (medical services) and/or treatment (medicines) for the vomiting?   - Borrow money to make the payment? - Sell an asset to make the payment? - Use money reserved for something else to make the payment? Please specify what the money was meant for originally: | Yes / No / Don’t know  Yes / No / Don’t know  Yes / No / Don’t know | 1 / 2 / 99  1 / 2 / 99  1 / 2 / 99 |  |
| VM22 | How many times were you vomiting per day most times during the period that you were vomiting? | 1-2 times  3-4 times  5-6 times  7 and above  Don’t know | 1  2  3  4  99 |  |
| VM23 | How many times were vomiting per day at the most severe period of the vomiting? | 1-2 times  3-4 times  5-6 times  7 and above  Don’t know | 1  2  3  4  99 |  |
| VM24 | How long did this severe period last for? | Less than 1 week  1 week- 1 month  >1 month but less than 3 months  3 months and above  Don’t know | 1  2  3  4  99 |  |
| VM25 | Rate the overall severity of the pain/discomfort/distress of the vomiting throughout the period you were vomiting during the pregnancy. SHOW CARD. | A  B  C  D  E  F  G  H  I  Don’t know | 1  2  3  4  5  6  7  8  9  99 |  |

Note: As this study was part of a bigger project on maternal morbidity within communities in Yola, North-east Nigeria, only questions relevant to this paper’s focus have been provided.
