# Supplementary material for: Self-reported vomiting during pregnancy in North-east Nigeria: perceptions, prevalence, severity and impacts
Source: BMC Pregnancy Childbirth. 2022 Aug 4;22:614. doi: 10.1186/s12884-022-04916-4 (PMC9351193; doi:10.1186/s12884-022-04916-4)
Supplement: Supplementary file 3 — Additional file 3. Entire participant population in survey (n=640). [file 12884_2022_4916_MOESM3_ESM.docx]

## **Supplementary file 3: Entire participant population in survey (n=640)**

| **Characteristic** | **Frequency** | **Weighted Proportion % (95% CI)** |
| --- | --- | --- |
| **Residence**  Rural  Urban | 161  479 | 25.0 (8.0- 56.1)  75.0 (43.9- 92.0) |
| **Age (years)**  15-19  20-34  35-49 | 52  476  93 | 8.5 (5.3- 13.4)  76.7 (73.4- 79.7)  14.8 (10.8- 20.0) |
| **Type of marital union**  Monogamous  Polygamous | 475  147 | 74.8 (66.9- 81.4)  25.2 (18.6- 33.1) |
| **Religion**  Islam  Christianity | 476  161 | 74.7 (58.8- 85.9)  25.3 (14.1- 41.3) |
| **Literacy**  Can read in any language  Cannot read in any language | 255  341 | 44.2 (34.8- 54.0)  55.8 (46.0- 65.2) |
| **Main occupation**  Unemployed/house-wife  Unskilled  Skilled | 361  202  72 | 58.0 (54.3- 61.6)  31.3 (24.7- 38.9)  10.7 (6.6- 16.9) |
| **Highest educational level completed/currently attending**  Never attended school/ non-western education  Primary  Secondary  Post-secondary | 199  137  243  58 | 32.6 (23.6- 43.1)  19.4 (15.0- 24.6)  39.3 (30.4- 48.9)  8.8 (5.1- 14.9) |
| **Husband’s main occupation**  Unemployed  Unskilled  Skilled | 13  366  257 | 1.7 (1.0- 2.9)  59.7 (49.7- 69.0)  38.5 (29.4- 48.6) |
| **Husband’s highest edu. level completed/currently attending**  Never attended school/ non-western education  Primary  Secondary  Post-secondary | 141  51  246  187 | 24.0 (17.3- 32.4)  8.2 (5.7- 11.7)  38.4 (33.6- 43.4)  29.4 (21.6- 38.5) |

## 
